# 15 years of minimally-invasive glaucoma surgeries (MIGS) experience and data: a rationale for optimal clinical decision-making

**DOI:** 10.5935/0004-2749.2023-1004

**Published:** 2023

**Authors:** Tiago Santos Prata, Fabio Bernardi Daga, Ticiana De Francesco, Iqbal Ike K. Ahmed

**Affiliations:** 1 Department of Ophthalmology, Universidade Federal de São Paulo, São Paulo, SP, Brazil; 2 Department of Ophthalmology, Mayo Clinic, Jacksonville, Florida, USA; 3 Glaucoma Sector, HMO, Opty Group, Brazil; 4 Hospital de Olhos Leiria de Andrade, Fortaleza, CE, Brazil; 5 University of Toronto, Toronto, Ontario, Canada; 6 John Moran Eye Center, University of Utah, Salt Lake City USA

Even though the pathophysiology of glaucomatous optic neuropathy is not fully understood, elevated intraocular pressure (IOP) remains the most important modifiable risk factor for glaucoma development and progression^(1,2)^. Therefore, effective continuous IOP reduction is the primary goal of the medical and surgical treatment of glaucoma^(3)^.

Although traditional incisional glaucoma surgeries lower IOP substantially, most rely on more invasive approaches with a considerable rate of complications and difficult postoperative recovery^(4,5,6)^. Consequently, in the past, glaucoma surgery was typically reserved for patients with advanced glaucoma on “maximally tolerated medical therapy” or those with progressive disease who were at high risk of severe vision loss. Over the past two decades, new, more physiologic surgical procedures involving less tissue manipulation, faster recovery, and lower complication rates than conventional filtration procedures have been developed, resulting in a substantial shift in the surgical approach to glaucoma^(7)^. These minimally invasive glaucoma surgeries (MIGS) may be categorized based on their different surgical targets: trabecular meshwork bypass, suprachoroidal space drainage, or bleb forming procedures^(8)^. Bleb-forming MIGS are more efficient at lowering IOP than other types of MIGS but increase the amount of surgical dissection and risk. We prefer to regard these procedures as microinvasive bleb surgery (MIBS).

The efficacy and safety of MIGS have been extensively investigated over the past few years^(9,10,11,12,13,14,15,16)^. However, considerable variability and uncertainty remain worldwide regarding their effective application in clinical practice, leading to suboptimal patient selection and outcomes. We believe this also to be an issue in Brazil. Therefore, the present study aims to address some misconceptions about MIGS and provide a framework to produce more practical guidelines for decision-making based on a critical analysis of MIGS literature and expert opinions. In this article, we will provide more emphasis on the procedures currently available in Brazil.

## BRIEF DESCRIPTION OF AVAILABLE MIGS OPTIONS

Various MIGS procedures have been described over the past 15 years^(17)^. In general, this group of novel techniques may sufficiently lower IOP and the medication burden to delay or minimize the need for conventional incisional surgeries. The favorable safety profile of these procedures allows treatment during the early stages of glaucoma (mild to moderate open-angle glaucoma patients) with minimal impact on possible future filtering surgery. MIGS can also be combined with cataract surgery, making them a valuable option for glau comatous patients with coexisting symptomatic cataracts and reducing the glaucoma medication burden. The addition of MIGS to cataract surgery has reduced the rate of major secondary surgery and the progression of visual field defects^(9)^.

As aforementioned, we will address the main Schlemm’s canal-based MIGS alternatives available in Brazil. These procedures can be subdivided into trabecular bypass stent (iStent; Glaukos Corporation), ab-interno canaloplasty (iTrack microcatheter; Nova Eye Medical), ab-interno excisional goniotomy (Kahook Dual Blade; KDB, New World Medical), and gonioscopyassisted transluminal trabeculotomy (GATT). These Schlemm’s canal-based procedures divert aqueous flow directly to the Schlemm’s canal, thereby bypassing most of the outflow resistance (approximately 50%-75% of the outflow resistance lies within the trabecular meshwork and the inner wall of the canal)^(3)^. Due to physiologic episcleral venous resistance, IOP-lowering has a lower limit - typically low to mid-teens^(18)^. Furthermore, secondary increased resistance in the distal outflow system may occur, especially in patients with advanced glaucoma^(19)^. Due to these considerations, Schlemm’s canal-based MIGS are of limited value for advanced glaucoma patients requiring significant IOP reduction^(2)^. Therefore, conventional and bleb-based incisional glaucoma surgeries are still important, especially in cases of advanced disease. MIGS are not necessarily designed to replace them but to fill the existing gap between drops or laser treatment and traditional incisional surgeries.

## CHARACTERIZATION OF MIGS-RELATED DATA

Over 700 articles have been published on MIGS so far. A preliminary review reveals different study profiles when comparing the available data on the different types of MIGS. They include several industry-sponsored, high-cost multicenter trials with numerous participants and robust designs (many are randomized clinical trials). This is often used for studies with device implant-based MIGS, which require greater regulatory scrutiny. These characteristics can have some limitations, such as financial interest and the adoption of specific inclusion/exclusion criteria, which can sometimes make it challenging to validate the results externally. On the other hand, there are smaller, often non-comparative retrospective non-sponsored studies, with shorter follow-up times and less robust designs. They usually include patients with a more diverse clinical profile and may provide a broader discussion regarding their findings. These smaller studies are found for both device-based and non-device implant-based MIGS.

The studies investigating Schlemm’s canal-based MIGS procedures are mostly focused on trabecular bypass implants, which increases the availability of iStent-related data compared to the other procedures. Overall, studies on Schlemm’s canal-based MIGS provide short to mid-term results regarding their efficacy and safety^(20)^. Though effectiveness may vary depending on the choice of Schlemm’s canal-based MIGS, study population, glaucoma type, success definition, and follow-up duration, success rates usually range between 75 to 90% at 12 months. Regarding safety profiles, side effects are typically transient (self-limited) and mild. Sight-threatening events are rare.

Very few prospective studies compare the outcomes of the different options of Schlemm’s canal-based MIGS. Therefore, it is not possible to provide an evidencebased statement as to which is more effective or has a better safety profile at this time. However, based on a critical review of the literature and our surgical experience, we believe there are trade-offs between efficacy and safety. Procedures that provide a greater magnitude of IOP reduction usually require more tissue manipulation and are most commonly accompanied by more adverse intraoperative and postoperative events. Finally, there is a paucity of data regarding costs, clinical utility measures, and quality of life, which makes it difficult to establish a clear and definitive comparison between Schlemm’s canal-based MIGS, medications, and/or conventional glaucoma surgery.

## RATIONALE FOR USE OF MIGS

At this point, we believe providing physicians with more practical guidelines is essential. The first question that must be answered during the decision-making process is, “Where is your patient in the glaucoma journey?” Is this a newly diagnosed patient? Undergoing cataract surgery? A patient with intolerance and/or poor compliance with the current medical regimen? Is IOP above target? Is glaucoma progressing despite the administration of maximum-tolerated medical therapy? Or is it a patient with previous failed glaucoma surgery? These different stages of the disease are directly related to the suitability of each surgical alternative and certainly impact our choice between MIGS and conventional incisional surgery.

In this context, not only general recommendations but also specific guiding principles should be considered, as they will allow individualizing each patient’s surgical option. The main factors that influence decision- making include lens status/cataract, diagnosis, severity, duration of disease, age, disease control (IOP, number of medications, and structure-function stability), compliance, tolerance/side effects with the current treatment, previous surgical procedures, costs, access, and level of invasiveness. It is important to remember that “controlled” glaucoma implies that the IOP measured in the office is on target, stays consistent 24 hours a day, and the patient is adherent. We cannot overemphasize how prevalent poor medication tolerance and adherence are worldwide and in Brazil^(21,22)^. For this reason, MIGS is considered in patients who may be controlled on anti-glaucoma medications but are at risk of glaucoma progression due to intolerance or poor medication adherence.

Given these considerations, we believe that the most common indications for employing Schlemm’s canal-based MIGS procedures for managing open-angle glaucoma patients would be: (A) Patients with mild to moderate glaucoma with controlled IOP requiring a better medication regimen due to side effects or poor adherence with or without previous selective laser trabeculoplasty or (B) patients with an indication for cataract surgery presenting with mild to moderate controlled glaucoma; in scenarios A and B, the main focus is to reduce the number of glaucoma medications; (C) phakic or pseudophakic patients, or those with an indication for cataract surgery with ocular hypertension or mild to moderate glaucoma, whose IOP with medications is above the target: in these case, IOP reduction is the primary objective of the procedure.

As previously mentioned, it is essential to remember that the magnitude of IOP reduction with these procedures is limited compared to conventional bleb surgeries. For those surgeons adopting MIGS, we suggest starting with more uncomplicated and well-controlled cases (patients with mild glaucoma with well-controlled IOP, under few topical medications, and undergoing cataract surgery), in which the disease prognosis is not dependent on the outcomes of the MIGS procedure. We recommend expanding the surgical indications as the surgeon gains more confidence and masters the techniques.

Finally, it is also important to highlight the clinical situations in which we are less likely to recommend MIGS. We should avoid performing MIGS in advanced glaucoma patients with uncontrolled IOP due to the need to achieve low IOPs and the poor likelihood of success. As Schlemm’s canal-based MIGS offer a moderate magnitude of IOP reduction and there is a risk of IOP spikes in the initial postoperative period, we are cautious in recommending these for eyes with advanced glaucoma or for any patient (regardless of disease stage) who needs a greater IOP reduction than these procedures can provide. In some specific patients with advanced glaucoma, such as those with good IOP control and stable disease (based on functional and structural tests) who are undergoing cataract surgery, MIGS can be used to keep the IOP under control and reduce the medication burden.

The results available to date suggest that Schlemm’s canal-based MIGS are effective and safe alternatives for managing mild to moderate open-angle glaucoma, positively impacting IOP control and the number of medications. They may therefore address the needs of poorly adherent patients. Success rates are impacted by the proper surgical indication. Therefore, the knowledge of the characteristics and limitations of each procedure, in combination with an adequate assessment of each patient’s profile, is of utmost importance for optimal clinical decision-making. We believe more studies are needed to evaluate costs, clinical utility measures, and quality of life data.
